# A Spike-Timing Pattern Based Neural Network Model for the Study of Memory Dynamics

**DOI:** 10.1371/journal.pone.0006247

**Published:** 2009-07-24

**Authors:** Jian K. Liu, Zhen-Su She

**Affiliations:** 1 Department of Mathematics, University of California Los Angeles, Los Angeles, California, United States of America; 2 State Key Lab for Turbulence and Complex Systems and Center for Theoretical Biology, Peking University, Beijing, China; University of California, Irvine, United States of America

## Abstract

It is well accepted that the brain's computation relies on spatiotemporal activity of neural networks. In particular, there is growing evidence of the importance of continuously and precisely timed spiking activity. Therefore, it is important to characterize memory states in terms of spike-timing patterns that give both reliable memory of firing activities and precise memory of firing timings. The relationship between memory states and spike-timing patterns has been studied empirically with large-scale recording of neuron population in recent years. Here, by using a recurrent neural network model with dynamics at two time scales, we construct a dynamical memory network model which embeds both fast neural and synaptic variation and slow learning dynamics. A state vector is proposed to describe memory states in terms of spike-timing patterns of neural population, and a distance measure of state vector is defined to study several important phenomena of memory dynamics: partial memory recall, learning efficiency, learning with correlated stimuli. We show that the distance measure can capture the timing difference of memory states. In addition, we examine the influence of network topology on learning ability, and show that local connections can increase the network's ability to embed more memory states. Together theses results suggest that the proposed system based on spike-timing patterns gives a productive model for the study of detailed learning and memory dynamics.

## Introduction

Recurrent neural networks of the brain compute information through complexly spatiotemporal neural activity. Recent experimental observations and theoretical studies have proposed that spike-timing patterns (STPs) in the range of a few hundred milliseconds play a fundamental role in sensory, motor and high-level cognitive behaviors such as learning and memory [1]–[4]. For instance, songbirds, one of the most studied neural systems, learn and memorize the crystallized song composed by precise individual syllables as STPs [5]. Traditionally, firing rate is used to describe the activity of single neurons and neural networks. However, a memorized song as a STP contains not only firing activity, *i.e.*, whether neurons fire or not, but also firing timings, *i.e.*, when neurons fire. Therefore, memory has to be both reliable in firing and precise in timing. Firing rate as a average measure is reliable but not precise [2]. Thus the question is how to capture the precise timing of memory from STPs.

Recent experimental data of hundreds of spike trains from multi-electrode recording have identified repeated or periodic STPs [2]. There is a great interest in such a STP code in neural circuits. Neurons *in vitro* produce a STP in response to an external stimulus. However, neurons *in vivo* are modulated by local oscillatory neural activities and top-down inputs. In a cortical circuit, precise STPs thus reflect the interaction between internally generated activity and sensory information. On the other hand, memory states are global dynamical behaviors of the cortical network emerged from relatively simple neural and synaptic dynamics. It has shown that several different dynamical regions for the spontaneous network activity generated by Poisson background inputs can be identified [6]. However, dynamical behavior of neural networks in response to external stimuli is less well studied due to the difficulty of the mathematical description of nonlinear high dimension dynamical system [7], [8]. Thus the essential question is how to construct a global description of network states in terms of STPs, which is less dependent of the existence of background spiking noise and external inputs.

In this work, we address these questions by simulating a two time-scale biologically realistic neural network with dynamics evolving at two time-scales: the fast scale of neurons and synapses and slow scale of homeostatic presynaptic-dependent synaptic scaling. After training, the network converges to a stable state with a spare neural trajectory as a STP. By proposing a state vector for the STP induced by each stimulus, we show the distance of state vectors can be used to characterize learning process and several important phenomena of memory dynamics: partial memory recall, learning efficiency, learning with correlated stimuli. Specifically, we examine the influence of network topology on leaning ability, and show that local connections can increase the network's ability to embed more memory states. We also show that distance measure can capture the timing difference of memory states formed in partial memory recall tasks and correlated-stimuli learning tasks. However, firing rate and correlation coefficient fail to differentiate these similar memories. Together theses results suggest that the proposed system based on spike-timing patterns gives a productive model for the study of detailed learning and memory dynamics.

## Methods

### Neuron dynamics

The single neuron was modeled as a integrate-and-fire neuron [9], in which the membrane potential *V* was described when 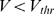 as:
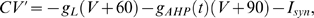
(1)where membrane time constants were 10 ms for all excitatory (E) and inhibitory (I) neurons (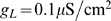; 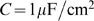). Neurons were heterogeneous in the sense that firing thresholds *V_thr_* was set from a normal distribution (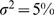 of the mean) with the mean for the E(I) cells as 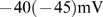. When *V_thr_* was reached at the spiking time *t_spk_*, *V* was set to 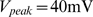 for the duration of the spike (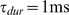). Then after the spike, *V* was reset as the repolarizing potential 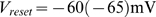 for the E(I) cells, at the same time, the afterhyperpolarization 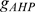 was turned on and changed as 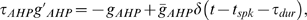 where 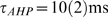 for the E(I) cells. The Dirac function *δ* was used to set a stepwise increment of 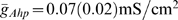 for the E(I) cells whenever a spike occurred. The refractory period 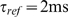 for all neurons.

### Short-term plasticity

Short-term plasticity was incorporated in all synapses and modeled as [10], [11]:
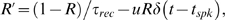
(2)

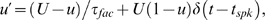
(3)where *R* (*u*) was the short-term depression (facilitation) variable with the time constant 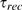 (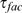), and subjected to the pulsed decrease 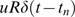 (increase 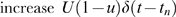) due to the spike at 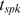. The cumulative synaptic efficacy at any time was the product *Ru* that was incorporated into single synaptic dynamics below. Specifically, 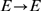 synapses exhibited depression: 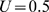, 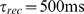, 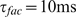; 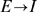 synapses exhibited facilitation 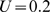, 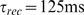, 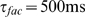. All inhibitory synapses exhibited depression as basket cell synapses [12]: 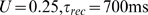, 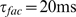.

### Synapse dynamics

Each neuron received four possible synaptic currents:
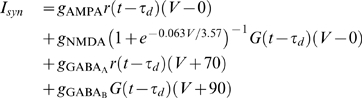
(4)where synaptic delays were uniformly distributed 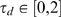. The receptor activation 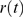 for fast AMPA and 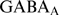 dynamics followed two-state kinetic models [13]:
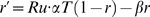
(5)where 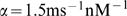 and 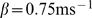 for AMPA; 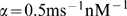 and 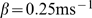 for 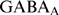. 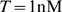 is the presynaptic transmitter concentration. NMDA and 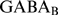 with slow dynamics were modeled as [14], [15]:
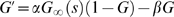
(6)

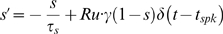
(7)

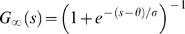
(8)where for NMDA: 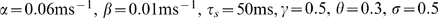; for: 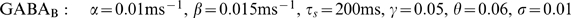. In all synapses, *Ru* was included for the short-term plasticity. The ratio of NMDA to AMPA synaptic weights was fixed as 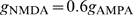 for all E-cells. The ratio of 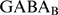 to 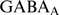 synaptic weights was fixed as 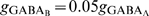 for all I-cells.

### Homeostatic Synaptic Scaling

Here we used a modified presynaptic dependent synaptic scaling (PSD) [9], which assumed that the change of synaptic weights was dependent on the activities of both pre- and post-synaptic neurons. When a discrete system was considered, synaptic weights from cell *j* to cell *i* at trial *τ* was denoted as 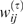. Then the PSD rule read
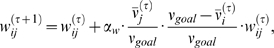
(9)where 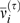 was the average activity at trial *τ* for cell *i*, which was given by
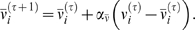
(10)The learning rate was 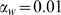, and the time scale of activity 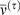 was 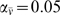. 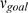 was the target activity set as 1(2)Hz for E(I)-cells. Therefore, learning dynamics and neural dynamics were coupled via 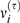, the instantaneous firing rate of cell *i* at the *τ*th trial, was defined as
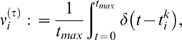
(11)where 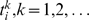 was the spiking time of cell *i* within the trial *τ*, and 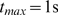 was the running time of one trial. When a periodic stimulus was used, one was equivalent to one period. The mismatch between the instantaneous and average firing rate adjusted the interaction between neural activities and learning dynamics until the network reached a stable state where 
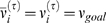
. One distinguished feature of homeostatic synaptic plasticity is that a rather long history of activity is considered with a large time constant. In particular, Eq. 10 and Eq. 9 define a dynamical system with a mutual coupling of two distinct processes, in which neural dynamics and learning dynamics interact with distinct timescales. This results in a complex evolution since neural dynamics depends on synaptic weights of learning dynamics and learning dynamics evolves according to activity of neural dynamics.

### Network input

A stimulus was composed by randomly selected 10 E- and 5 I-cells that fired 1 Hz. The spiking time of input was assigned at 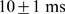 (mean±SD) following a normal distribution relative to the onset of each period of 1 s, thus one subset of cells was firing at the beginning of each period. Selected input cells were activated by a 1 Hz excitatory postsynaptic current. Qualitatively similar results were obtained when the SD of the Gaussian time window was increased. We used a small SD to simulate a brief highly synchronous input to the network [16].

### Model Networks and Parameters

All simulations were done for a network with 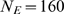 E- and 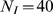 I-cells connected with a probability 10% between any pair of cells based on experimental data [17]. Results were robust to the network size and connection probability. Initial synaptic weights were chosen from a normal distribution with the mean as 0.6 nS for excitatory and 0.01 *µ*S for inhibitory synapse, respectively, and the SD as large as 200% of the mean to introduce the heterogeneous distribution. If initial weights were chosen as non-positive, reset them uniformly within the range of twice of the mean. As a result, most of initial weights were weak. Results are qualitatively same with the different setup of initial weights as Poisson distribution. To avoid the plasticity to induce the unphysiological state where a single presynaptic cell fired a postsynaptic cell, the maximal excitatory synaptic weight were set as: 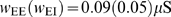. All inhibitory weights were fixed without plasticity. All simulations were done in C++ using the explicit Euler method with a time step 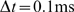. The code can be downloaded at the author's web page (∼http://www.math.ucla.edu/~liujk/publication/index.html)

## Results

### State Vector and Learning Time

**Figure 1:**
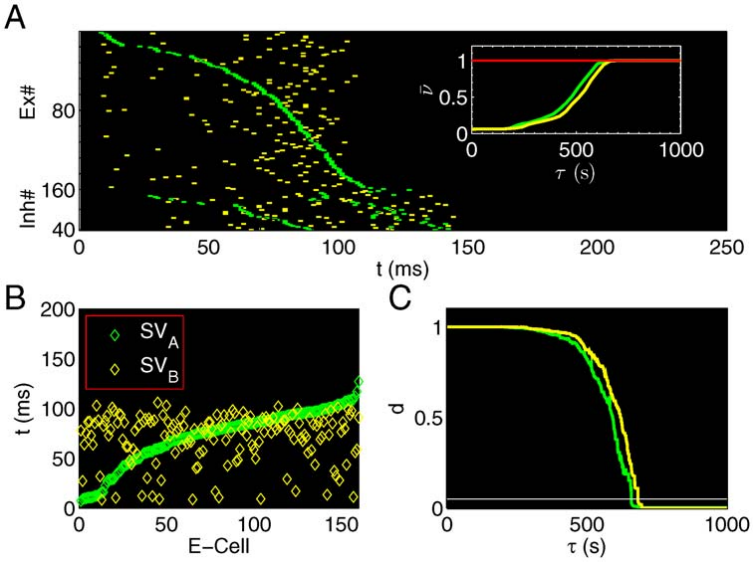
Spatiotemporal activity patterns are developed during the learning. (A) STPs are induced by stimulus A (green) and B (yellow) in the same coordinated neural space sorted in the ascending order of E-cells' firing time with respect to stimulus A. The inset shows that both average firing rates are convergent, 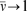. (B) State vectors 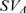 and 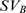 induced by stimulus A and B, respectively, are in the same neural space formed by 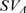; (c) Learning time 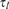 is defined as the minimal time such that the normalized distance *d* falls below the horizonal white line 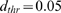. Here 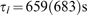 for stimulus A(B), respectively.

The current network has a feature that an unique stable trajectory of E-cells can emerge at the end of a learning process (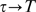, where 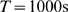 throughout the whole study). In this final state, every E-cell fired only once within one period [Figure 1(A), trajectory A (green) induced by stimulus A (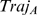)], and 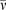 and synaptic weights reached a steady state, 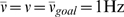 for E-cells as in Eq. 10.

By sorting all E-cells according to their firing time (during any specific learning process), and using their ordered cell indexes to define a 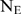-dimensional vector, one can obtain a 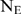-dimensional state vector (SV) 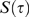 as:
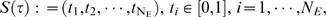
(12)where 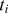 was the firing time of the *i*-th E-cell sorted in the ascending order [Figure 1(A), 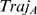 (green)]. 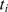 was bounded within one period of the short-term evolution, 1 second throughout this study, and set as 0 if a cell did not fire. Note that initially, only a portion of the E-cells fire, so that *SV* is not of full rank. As the learning process proceeded, more cells fired as synaptic weights were updated. Thus, *S*(*τ*) evolved to a state vector of full rank 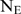, which then characterized a STP. Hereafter, we denoted 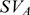 as the characteristic state vector at the end of each learning process with a given stimuli *A*:
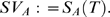
(13)In practice, *S*(*τ*) stably converged to 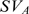 at a finite time 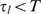, which can be termed the learning time 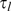. The practical definition of 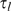 can be achieved using the average distance (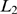 norm) of two vectors: 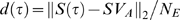 [Figure 1(C)]. Note that in the stable case where the maximum distance is *d*(0), let 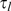 be the minimum time such that the normalized distance for all time 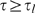 was less than a threshold 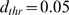 chosen throughout this study to insure 95% accuracy, *i.e.*,
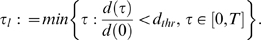
(14)Therefore, with the assumption that each memory corresponded to a STP, the spike timing was the only important variable. With different stimuli, the network presented different *SV* s, which represented different memories [18]. One can study the simulated memory dynamics of neural network with their characteristic STPs, in which *SV* s were used to measure the similarity between different STPs. To compare different STPs, one need to compare SVs in the same coordinate system, therefore, only one set of ordered cell indexes was used to calculate different *SV* s. As shown in Figure 1, the second 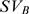 (yellow) was coordinated into the same neural space formed by 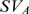. The distance of two vectors was calculated to compare the similarity of two STPs, which became a global description of two network states.

### Network Topology and Learning Ability

It has been shown that synaptic connections among neurons in the local cortical network are not random but with a higher probability between nearby neurons [17]. To define the network topology, all E-cells were arranged into one dimensional space as a ring since the periodic condition was used. I-cells provided the global inhibition to all randomly selected E-cells. There are three common topologies used in literatures:

1.) Uniformly connection (UC): each neuron connects with its neighbors with a probability from the uniform distribution. Then the space range of connections for a postsynaptic neuron *i* at the position 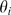 can be characterized by a diameter *diam* such that neuron *i* connects with other neurons *j* at
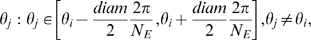
(15)where self-connections are excluded. In this way, there is a natural relationship between *diam* and the connection probability *p* as 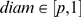. Rigorously speaking, 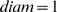 is a special case since 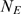 is even and both boundaries are coincided due to the ring structure. In practice, it is well defined since multiple connections between a given pair is avoid. 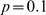 is used in our work [17]. It is easy to recognize two extreme cases: one with global random connections as 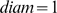, and the other with highly localized connections as 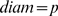.

2.) Normal connection (NC): each neuron connects with its neighbors with a probability from the normal distribution. Then the space range of connections for a postsynaptic neuron *i* at the position 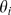 can be characterized by the standard deviation (SD) *σ* of normal distribution such that neuron *i* connects with those neuron *j* at the position with the mean as 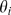 and the SD as *σ*. In this way, the variation of 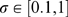 gives the global or local space range of connections, which is comparable with the UC case, but not same.

3.) Small world connection (SC): this case is based on the NC case but with one more condition: there are 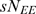 synapses for each neuron to connect randomly and globally with all other neurons, where 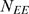 is the number of presynaptic neurons for each postsynaptic neuron. In our work 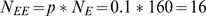. In this way, there are some percentage of long-range connections within the network that is dependent of the value of 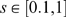. Essentially, this type of network is a small world network. It should be noted that there are two parameters to describe the SC network: 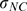 characterizing the degree of local connections (for the clarification, denote 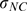 in the SC network, and leave *σ* to refer the NC network), and *s* characterizing the degree of long-range connections.

**Figure 2:**
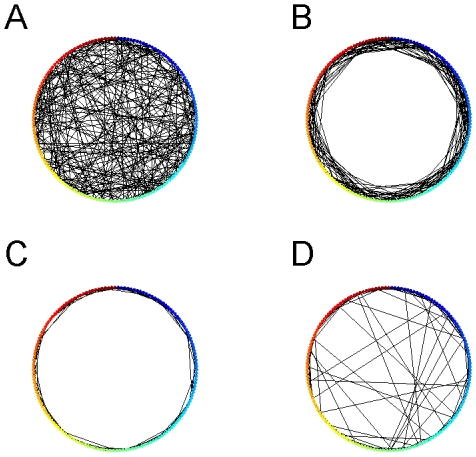
Visualization of three different network topologies. (A) UC network with 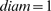 is a global random network. (B) UC network with 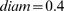. (C) NC network with 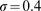. (D) SC network with 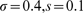. E-cells are colored and labeled along a ring. Black solid lines between E-cells are excitatory synapse connections. Only 10% of total synapses are shown. There are 160 synapses in each panel.

**Figure 3:**
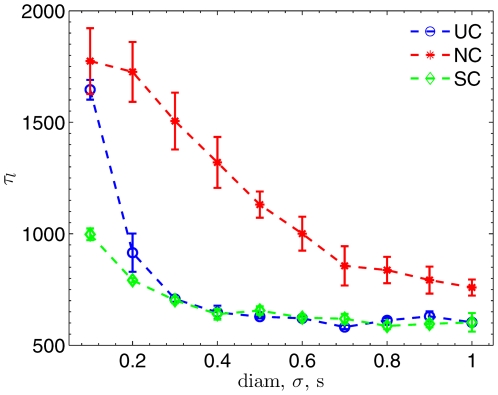
Learning time decays with the fading of network topology. Learning time 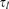 decreases when network become more random by increasing topology parameters (blue: UC with *diam*; red: NC with *σ*; green: SC with *s* and 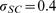). Note that the increasing *diam* and *s* make the UC and SC network similar, where the optimal learning dynamics reaches at an immediate connection radius 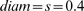. Error bars (S.E.M.) are calculated with 3 simulations with different random number seeds.

Figure 2 shows four typical networks with different connection topologies. In all panels, the number of synaptic connections was same, however, the degree of localization and globalization were varied with different topologies. All four networks have been suggested to exist in the cortex [19]. To understand how the variation of topology determined the learning time, we systematically changed network parameters 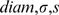 within the range 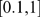 to compare the learning time 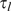. As shown in Fig. 3, 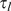 with the same stimulus A as in Fig. 1 decreased with the increasing of 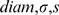; this was a consequence of the fact that the propagation of activity became significantly slower as connections became more local [9]. This effect can be understood from the learning rule Eq. 9 that is presynaptically dependent. Whenever the presynaptic cell was a firing input, its action potential only propagated to the downstream postsynaptic cells in its neighborhood. Therefore, activity can not spread to the whole network until its neighbors fired, which resulted in that the network highly localized with 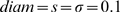 used the largest 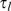 to reach the target. It should be noted that 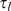 reached its asymptotical value at around 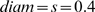, which implied that the network with an immediate connection diameter reached to its optimal learning dynamics.

Another notable phenomenon indicated in Fig. 3 is that the learning time in the small world network (SC: 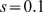, 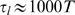) with sparse long-range synaptic connections was much less than that in other two network topologies (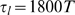 in UC with 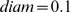 and NC 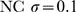). This type of network, which plays a similar role as a small-world network that has been exemplified by the neocortex [19], can dramatically reduce the average path length, and is critical for globally distributing the results of local computation throughout the entire cortex.

### Memory Recall

**Figure 4:**
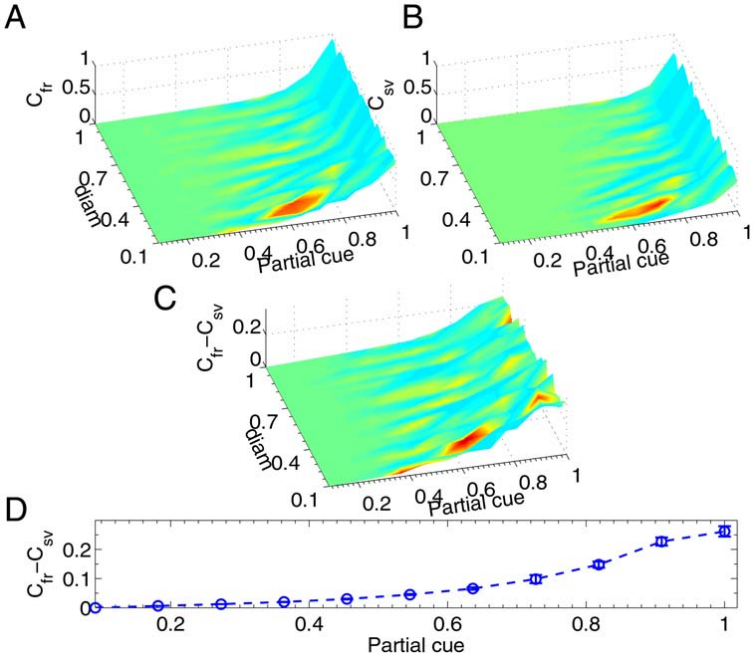
Network in response to partial cues shows unreliable memory with the distracted spike timings. (A,B) Network response measured with firing rate 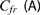 (A) and distance 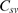 (B) as a function of the intensity of partial cue (E-input) and connection diameter. Both are normalized and 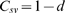 to compare with 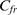. (C) Overestimation of recalled memory 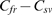 are the subtracted matrix (B) from (A). (D) Average 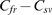 over all values of *diam* increases with the intensity of partial cues. Error bars (S.E.M.) are collected from 3 stimulations with different random number seeds. Each data point is an average result of the variation of the intensity of I-inputs.

The STP described by *SV* was a natural characteristic of the memory state. With *SV*, one can characterize the memory recall process in terms of the distance between STPs instead of firing rate [20]. In general, memory recall is assumed as a process of the full memory recovery. Here it was described by the intensity of the response to a fraction of E-inputs previously memorized (partial cues), then we studied this process by varying the number of stimulated E-cells (E-inputs) after the whole stimulus was learned. This process was designed as follows. Let the network evolved first with the full cue (the whole set of stimulus) until it reached its steady state, then learning was turned off since synaptic weights were stabilized, and we increased the intensity of E-inputs stepwise as the partial cues of associative memory [20]. The changing of the number of inhibitory inputs had little effect comparing with E-inputs, thus the result below were averaged over all fractions of inhibitory inputs. Figure 4(A,B) shows the evolving of the recall function with different partial cues, where two measures of recalled memory quality were calculated,

(i) the reliable measure
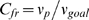
(16)was defined as the normalized firing rate, where 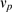 was the firing rate of partial cue response calculated over all cells, *i.e.*, 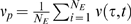 at any time *τ* (here we used 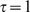 since learning was turned off after memory was formed and 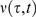 did not change with *τ*). 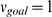 was the firing rate of full cue response.

(ii) the precise measure
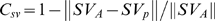
(17)was defined as the normalized *SV* distance, where 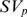 was the state vector of partial cue response, and 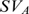 was the state vector of the reference state in Fig. 1(B). Choosing the reference state was important since the reference coordinate system (network space) had to be same to compare timings.

Both 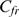 and 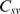 were consistent in that they showed the same tendency similar to the input-output response functions observed in experiments [21]. However, the difference between two recall functions was that 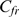 showed higher response than 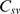 [Fig. 4(C,D)], and the overestimation 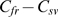 increased with the increasing of the intensity of partial cues, and reached the maximum at the full cue where the fraction of E-inputs was 1. At the full cue, both 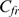 and 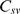 should reach to their ideal value 1, which meant that the full memory was recovered. However, 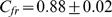 and 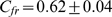. This deviation was due to the effect of the variation of inhibitory inputs. Inhibitory perturbation had a small effect on firing rate, but a great impact on the precise timing of STP. We should stress that after memory was formed by the PSD learning, network states in response to partial cues was only dependent on the balance of excitation and inhibition. A given partial cue induced some cells fire but not at the exact time as in the full cue case. Such a timing difference of recalled memory may become significant in the learning process requiring precise temporal information, such as speech signal patterns [22]. If thousands of memory states are embedded within the brain, the timings of STP are more sensitive to be distracted than the firing activity, which is an evidence for temporal coding rather than rate coding [23]. In reality, the same neural group could fire at different time to represent different STPs, then the only difference in these memory states is their timings not firing activities. This supports the idea that differential timings of spikes might be a biological mechanism to increase the memory capacity [4].

### Learning with Correlated Stimuli

It has been shown that a single neuron is sensitive to correlated inputs [24], we studied the similar question of the effect of correlated inputs to network behaviors. If we consider a speech letter ‘A’ with numerous memorized versions from different persons, and take the standard version as the reference state, all other versions can be composed into a correlated space. How to characterize the similarity of memories induced by these different versions of ‘A’? It is reasonable to assume different versions induce the same (or similar) group of cells to fire, thus they have the same firing activity. Consequently, the only difference has to be in their memory timings, which can be described by the distance of different *SV* s, but not firing rate. Here we described two correlated stimuli with an correlation index 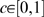 according to the number of cells coexisted in both stimuli, then we obtained a series of STPs 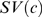 as a function of correlation index *c*, and studied these correlated memories in terms of different STPs at the network level with the effect of network topology by measuring their correlation coefficients 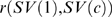 and distances 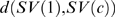 between the reference state 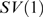 and evolutional state 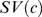 with different *c*.

**Figure 5:**
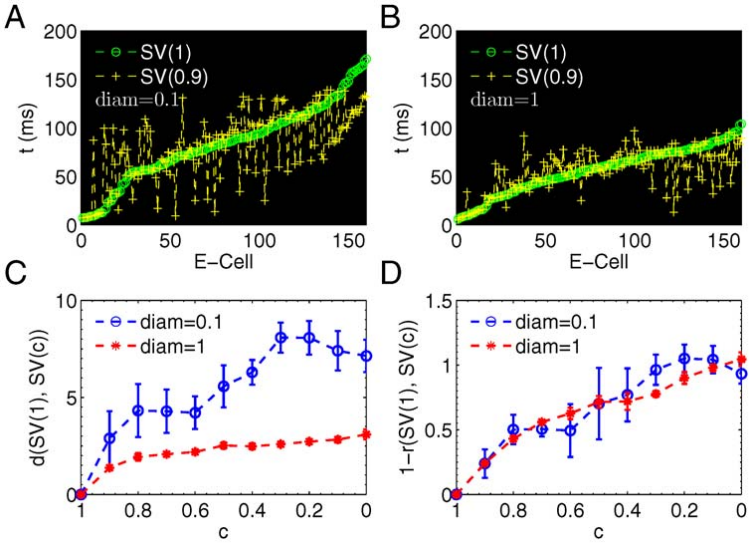
Memories induced by correlated stimuli show distinct spike timings. Learning correlated stimuli generates distinct memories with different spike timings but the same firing activity. (A) State vectors induced by the reference stimulus (*c* = 1) and a correlated stimulus (*c* = 0.9) show large different timings in a network with 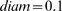. (B) State vectors induced by the reference stimulus (*c* = 1) and a correlated stimulus (*c* = 0.9) show small different timings in a network with 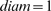. (C) SV distance becomes significantly larger when 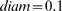; (D) Correlation coefficients *r* nearly overlap to each other. Here 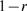 is used to compare the case with *d*. In (A) and (B), 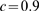 means that there is only one different input cell within the stimulus consisted of total 10 input cells. Therefore, the difference between 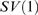 and 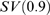 is due to only one unshared input cell.

Fig. 5(A) presents typical state vectors with different *c* in a locally connected network with 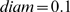. Here we showed two memory states induced by two correlated stimuli that were consisted of 9 shared and only 1 unshared input E-cells, which generated two state vectors at 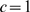 and 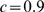. The state vector 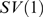 (green) of the reference state and 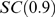 (yellow) of the correlated stimulus were quite different in their timings. Such a large difference of memory states was resulted from the small difference in two stimuli with only one distinct input cell. Similarly, Fig. 5(B) exhibits two memory states induced by the same stimuli but in a globally connected network (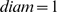). Note the timing difference of state vectors was smaller than that in Fig. 5(A), which suggested that the network topology had an effect in the learning of correlated stimuli. We provided a whole picture with the full range of the variation of stimulus correlation in Fig. 5(C,D). As shown in Fig. 5(C), 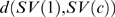 increased with the decreased correlation index *c*, which was a consequence of the stimuli-learning-memory dependency: distinct input stimuli generated distinct STPs. The learning behavior of the globally connected network with 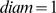 was dramatically different from that with the local connected topology with 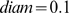. The distance 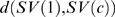 was doubled or more with local connections, as a result, memory states were significantly different. For a correlated stimulus, the distance between its memory state and the reference state was large when 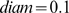, which suggested that local connections can enhance the network to clarify the specificity of a stimulus by holding distinct memory states. This may be important for the mechanisms of discrimination between correlated memory states during the learning of similar input stimuli. Back to the example at the beginning, the larger distance between correlated ‘A’ will make ‘A’ more sensible and easy to be clarified. Given the sensory system is a bounded space, the locally connected network has a large sensible space to memorize more signals. In this sense, the local connected topology is analogous to the brain ssytem with different and local topographic areas.

In contrast, correlation coefficient 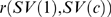 had a similar change independent of topology (Figure 5(D)), even though they captured the change over correlated stimuli. In other words, *r* was too insensitive to discriminate memory states. Therefore, with a given set of correlated stimuli, the dissimilarity of STPs characterized by *d* was more significant than those by *r*, and the distance of state vector was more powerful to detect the subtle changes of STPs.

## Discussion

### Learning dynamics

Here we employed a recurrent neural network model with two-scale dynamics, where the learning dynamics was slower than the fast dynamics of neurons and synapses. When a periodic stimulus was presented in the network, we obtained a simple neural trajectory after learning. The trail-based learning dynamics used in this study can guarantee that the characteristic timescale of synaptic plasticity is comparable with the biological timescale of experiments by setting the period arbitrarily long. The similar scheme has been used perviously in different contexts [25]–[27].

The current system could be generalized to a multi-scale dynamics for neural networks, in which synaptic dynamics occurs as a cascade of states with different levels of plasticity through metaplastic transitions [28]. In addition, the current network could be extended to include other types of synaptic plasticity at different time scales [29]. One example is the so-called reward learning based on the effect of neuromodulation on synaptic regulation, for instance dopamine [30]. Future work is needed to systemically study multi-scale learning dynamics of neural networks.

It should be noted that here we only consider the homeostatic PSD plasticity, which is one type of rate-based learning rules, where the change of synaptic weights is only dependent on the firing activity. There is another type of synaptic plasticity, so called the spike-based learning rule [31], which takes into account of the spiking timing to change synaptic weights. STDP, as a typical rule of this class, has been studied intensively [32]. One would expect that STDP can enhance the precision of the spike timing in STPs. However, it has been shown that the stable neural trajectory can not be developed if we only consider the STDP learning dynamics [33]. The future study need to consider the effect of several rules simultaneously, for instance the combination of PSD with STDP, or any type of rate-based and spike-based learning rules.

### Computational Ability of Spike-Timing Patterns

Although current technologies allow to record simultaneously hundreds of neurons with the precise spike timing, a theoretical framework for understanding and decoding the functional role of these large-scale STPs is still immature [2]. Here we used the state vector of STP as a precisely defined measure to characterize network states in the context of memory dynamics. With different stimuli, the network can embed those input information, propagate them within the whole network, and develop distinct STPs with information on both firing activity and timing. We have shown that the traditional measures, such as firing rate and correlation coefficient, can not capture the timing difference of memory states. One may question that this is because that memory is formed by the PSD learning, which is one type of rate-based learning rule without taking into account of the spike timing. However, this question is a consequence of the debate between coarse coding and fine coding [34]–[37]. Results in this work support recent theoretical and experimental observations about the significance of STPs in cortical networks [1]–[4]. We suggest that spatiotemporal patterns in local connected networks have an advantage to hold more memory states and be more sensitive to correlated stimuli, which has a same meant as the theory of neuronal group selection [38] where structured neuron groups evolve with time to generate different functional areas of the brain [4], [39].

The potential information stored in STPs is suggested to be larger and more efficient than that contained in firing rate patterns [2]. It has been proposed for several decades that brain's computation relays on the ensemble activity of neural groups at the population level [40]. In order to simplify the complexity of STPs in the present study, each neuron fire once without any background stochastic spiking activity. In the realistic cortical cortex, when the same stimulus presents repeatedly, the resulting STP is usually neither precise in timing nor reliable in firing rate when they are aligned to the stimulus onset [2]. Here we only focused on one ideal STP, however, future work has to be done with more complex STPs associated with spontaneous neural activity. Typical background activities are various rhythmical oscillations with different frequencies, which might also contribute to high-level cognitions [19], [41]. Therefore, it is important to address the question how the precise and reliable STP might emerge in the environment with dominating rhythmic oscillations.

### Characterizing Network States

Mathematical tools to describe high dimensional network states, even a few hundreds of neurons, are still demanding. Here we proposed a global description of network dynamics with a simple state vector, which could be extended to describe more complicated network states. A recent study proposed that the simple state vector can be extracted from the varying STPs when firing activity patterns were averaged over many trials [18], where the neural trajectory can be obtained by sorting with the latency of the peak firing rate. As in the current study, the experimental trajectory was a state vector with one particular temporal order. The difference is that our state vector has the memory of input stimulus, in contrast, the state vector given in Ref. [18] was internally generated in a memory task. However, the internal generation of state vector has to relay on the past experiences of the memory task. There was no trajectory in the control task without any learning experiences. Furthermore, they showed that the state vector not only encoded the past memory, but also provided the future planning of behaviors. As in our case where different stimuli generated different memory states, different initial conditions can provide different trajectories in their memory tasks. Future study could explore learning mechanisms behind this kind of memory task. Given a sea of experiment data on various behavior tasks at a large-scale network level, the theoretical approach like the current one may have an advantage.
